# UNDERSTANDING CAREGIVER BURDEN IN DECISION-MAKING IN ALZHEIMER’S DISEASE AND RELATED DEMENTIAS

**DOI:** 10.1093/geroni/igae098.1303

**Published:** 2024-12-31

**Authors:** Brandi Ginn, Ignacia Arteaga, Alma Hernández de Jesús, Corey Abramson, Daniel Dohan

**Affiliations:** University of California San Francisco, San Francisco, California, United States; University of California San Francisco, San Francisco, California, United States; University of California San Francisco, San Francisco, California, United States; Rice University, Tucson, Arizona, United States; University of California San Francisco, San Francisco, California, United States

## Abstract

Alzheimer’s disease and related dementias (ADRD) are chronic conditions that involve changes or loss of cognitive and social function. People living with dementia (PLWD) undergo a gradual expansion in caregiving needs, and many ultimately spend a portion of their lives in institutional settings, including nursing homes or assisted living facilities. This study uses a comparative ethnographic approach to examine how PLWD and their caregivers make sense of and navigate transitions from home to institutional care. It combines three years of detailed field observations and 84 in-depth interviews in California and Maine. Our data illustrate how socioeconomic inequality, disparate neighborhood contexts, and shared understandings of aging shape tensions caregivers experience as they navigate whether, how, and when to relocate PLWD for whom they care. Our results show cultural narratives encouraging independence and non-institutionalization of older adults intersect with caregivers’ desires, structural positions, and local institutional resources – creating tensions and are mentioned alongside the feeling of being burdened. These experiences and feelings emerge gradually as the health of PLWD declines and caregivers become aware of unmet needs and their own resource constraints. We unpack how diverse caregivers make sense of the burden they feel, define the thresholds to make decisions, map community resources, and experience a range of emotions such as frustration, guilt, and grief throughout the process. This contextualized analysis of how caregivers navigate ADRD decision-making offers insights into the affective nature of the process and the caregivers’ fortitude.

